# Development of an Italian National Epidemiological Register on Endometriosis Based on Administrative Data

**DOI:** 10.3390/jcm13113087

**Published:** 2024-05-24

**Authors:** Alice Maraschini, Emiliano Ceccarelli, Manuela Giangreco, Lorenzo Monasta, Valerio Manno, Dolores Catelan, Giorgia Stoppa, Annibale Biggeri, Giuseppe Ricci, Francesca Buonomo, Giada Minelli, Luca Ronfani

**Affiliations:** 1 Statistical Service, Istituto Superiore di Sanità, 00161 Rome, Italy; alice.maraschini@iss.it (A.M.); emiliano.ceccarelli@iss.it (E.C.); valerio.manno@iss.it (V.M.); 2 Department of Statistical Sciences, La Sapienza University of Rome, 00185 Rome, Italy; 3 Institute for Maternal and Child Health—IRCCS Burlo Garofolo, 34137 Trieste, Italy; manuela.giangreco@burlo.trieste.it (M.G.); lorenzo.monasta@burlo.trieste.it (L.M.); giuseppe.ricci@burlo.trieste.it (G.R.); francesca.buonomo@burlo.trieste.it (F.B.); luca.ronfani@burlo.trieste.it (L.R.); 4 Unit of Biostatistics, Epidemiology and Public Health, Department of Cardiac, Thoracic, Vascular Sciences and Public Health, University of Padua, 35122 Padua, Italy; dolores.catelan@ubep.unipd.it (D.C.); giorgia.stoppa@ubep.unipd.it (G.S.); annibale.biggeri@unipd.it (A.B.); 5 Department of Medicine, Surgery and Health Sciences, University of Trieste, 34127 Trieste, Italy

**Keywords:** endometriosis, epidemiology, incidence, prevalence, population-based

## Abstract

**Background/Objectives**: Endometriosis is a female chronic inflammatory disease in which endometrial tissue develops outside the uterine cavity. It is a complex pathology, which significantly contributes to morbidity in premenopausal women, leading to chronic pain, infertility, and subfertility negatively impacting physical and emotional well-being and the overall quality of life. The public health burden of endometriosis remains elusive and challenging to determine, and this uncertainty can lead to inadequate healthcare services and treatments. The objective was to estimate the incidence and prevalence of endometriosis in Italy using the hospital discharge records database via a population-based retrospective study, nationwide between 2011 and 2020. **Methods**: From the National Hospital Discharge Database, we selected all admissions with a diagnosis of endometriosis (ICD-9-CM, codes 617.x), supported by the presence of a procedure code of laparoscopy or any other surgical procedure allowing for direct visualisation of the lesions. The main outcomes measured: incidence and prevalence of endometriosis were estimated for the entire 2011–2020 period and by individual year, analysing the time trend and variability in different geographical areas of Italy. **Results**: There were a total of 134,667,646 women aged 15–50 years with one or more hospitalisations for endometriosis in all Italian hospitals. The incidence of endometriosis in Italy during this period was 0.839 per 1000 women (CI95% 0.834–0.844), exhibiting a statistically significant decreasing trend over the years. A discernible north–south gradient was observed, with higher rates documented in the northern regions. The prevalence rate stood at 14.0 per 1000 during the same period, and a similar north–south geographical gradient was identifiable in the prevalence rates as well. **Conclusions**: The utilization of national-level hospital data enables the generation of incidence and prevalence data for endometriosis without variations in methods and definitions, facilitating the evaluation of temporal trends and regional comparisons. Understanding and quantifying this phenomenon is essential for appropriate healthcare planning in various Italian regions.

## 1. Introduction

Endometriosis (EMS) is a female chronic inflammatory disease in which endometrial tissue develops outside the uterine cavity. It is a complex steroid hormone-dependent condition, and its development is regulated by oestrogen and progesterone levels [1]. EMS significantly contributes to morbidity in premenopausal women, leading to chronic pain, infertility, and subfertility, negatively impacting physical and emotional well-being and the overall quality of life [2,3].

The aetiology of EMS is not well understood, and diagnosis usually requires imaging techniques or surgery for more complex cases [4]. Treatment of EMS includes the use of oral contraceptive pills, gonadotropin-releasing hormone agonists and surgery in women with hormone-resistant pain [1].

Underdiagnoses, diagnostic delays, and the lack of reliable data on the prevalence and incidence of this pathology have strong clinical and social implications. The public health burden of EMS remains elusive and challenging to determine, and this uncertainty can lead to inadequate healthcare services and treatments [5,6].

Epidemiological studies conducted over the past three decades have aimed to determine the incidence and prevalence of EMS. Most studies focused on specific populations with pelvic pain, infertility, or gynaecological conditions. However, there is a lack of data based on the general population. Notably, an Australian cohort study found a cumulative prevalence of clinically confirmed EMS of 6.0% by age 40–44, increasing to 11.4% when including clinically suspected cases [7]. In Europe, a Spanish study estimated an overall incidence of 1.61 per 1000 women aged 15–54 years [8]. A systematic review with a meta-analysis reported a pooled incidence rate of 1.36 per 1000 person-years. Many of the studies included concerned women of reproductive age (between 15 and 45–55 years), but the age was heterogeneous among studies, ranging from 10 to 80 years. The review highlights the methodological heterogeneity in defining and studying EMS [9].

In Italy, there are no national data on the incidence and prevalence. However, in the Friuli-Venezia Giulia (FVG) region, an epidemiological register was enacted in 2011 to estimate the incidence and prevalence of EMS, combining hospitalization and anatomopathological data from the regional automated centralized record system [10]. The incidence of EMS in women aged 15–50 years was thus estimated to be 1.1 per 1000 resident women, and the prevalence, estimated from the incidence, to be 18 per 1000.

The present study aimed to estimate the incidence and prevalence of EMS in Italy using the National Hospital Discharge Database (NHDB). Despite potentially underestimating the phenomenon by only counting hospitalized cases, this data source enables a national estimate and analysis of differences among various geographical areas within the country.

## 2. Methods

Hospitalisation data for the decade 2011–2020 were analysed using the NHDB [11], collected by the Minister of Health and managed by the Statistical Service of the Italian National Institute for Health. The NHDB collects data on all hospitalization episodes provided in public and private hospitals in Italy, including demographic data (e.g., age, sex, residence, level of education), characteristics of the hospitalization, and clinical features (main and concomitant diagnosis, diagnostic or therapeutic procedures coded according to the International Classification of Diseases, Ninth Revision Clinical Modification (ICD-9-CM)).

All admissions with a main and concomitant diagnosis of EMS (ICD-9-CM, codes 617. x), supported by the presence of a procedure code of laparoscopy or any other surgical procedure allowing for direct visualization of the lesions, were selected (Table 1), as recommended by the 2014 guidelines of the European Society of Human Reproduction and Embryology [12]. Women diagnosed with EMS in the previous 10 years were excluded, so as to identify only incident cases. Only women aged 15 to 50 residing in Italy with a reliable pseudo-anonymized identifying code were selected, given the need to identify newly diagnosed cases specifically. Data were extracted at both the national and regional levels.

### Statistical Analysis

Crude rates were calculated as the number of cases per 1000 female population aged 15 to 50 residing in Italy [13] with a 95% confidence interval (CI), assuming the binomial distribution. Rates were calculated for each year from 2011 to 2020.

Starting from incidence data, and as suggested in the FVG study [10], the prevalence was calculated considering that EMS most commonly affects women during their reproductive years and tends to decrease after menopause. If (prevalence) = (incidence rate) × (average duration of the disease), and EMS is a chronic disease that lasts from diagnosis until at least menopause, incident cases will continue to accumulate until women reach menopause. We thus depicted the declining prevalence situation after menopause under the simplified assumption that menopause begins following a Gaussian curve with a mean of 51 years and a 95% confidence interval of ±5 years (standard deviation = 2.5). To simplify, we assumed that all women would enter menopause between the ages of 45 and over, and converted the probabilities from the Gaussian distribution to follow this assumption. Lastly, to represent the decline, lacking real data on which to base our decline in prevalence, we arbitrarily decided that, starting at age 45, each year, 20% of women entering menopause would cease to have the disease.

A comparison was conducted in two regions: FVG and Puglia to validate the estimated incidence rate calculated by NHDB and understand how many cases are overlooked by this count due to not being hospitalized. In these regions, a local project integrated data from an administrative database with those from a pathological anatomy registry.

## 3. Results

**Figure 1:**
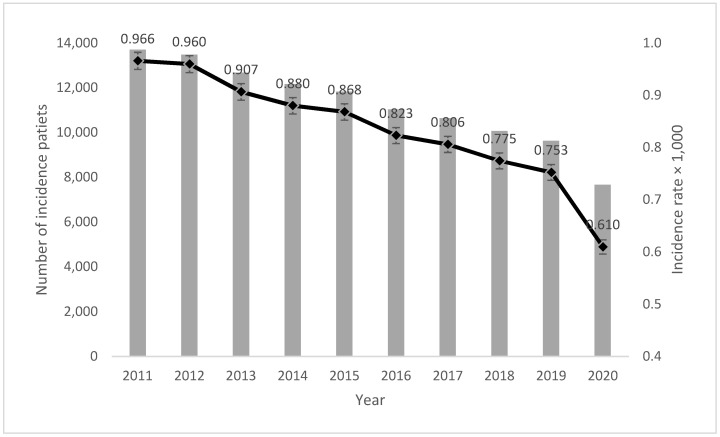
Annual temporal trend of endometriosis incidence during the 2011–2020 period.

**Figure 2:**
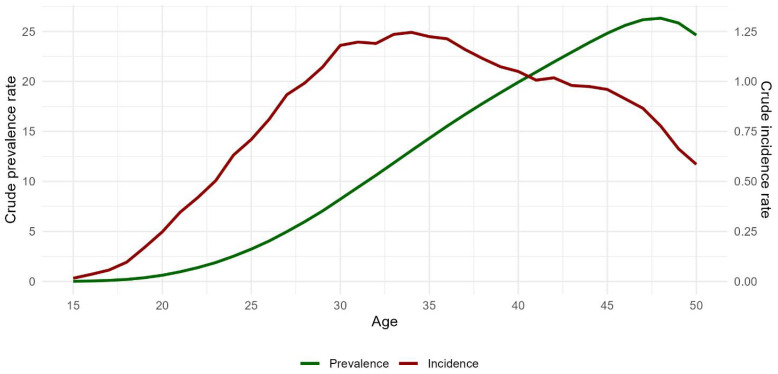
Age-specific incidence rate and prevalence of endometriosis during the 2011–2020 period.

During 2011–2020, 112,945 new diagnoses of EMS, confirmed through direct tissue visualization, were recorded for 134,667,646 women aged 15 to 50 resident in Italy. The incidence of EMS in Italy during this period was 0.839 (CI 95% 0.834–0.844) per 1000 women, displaying a significantly decreasing trend (*p*-value for trend <0.001), notably in the year 2020, likely due to the reduced access to healthcare services caused by the SARS-CoV-2 pandemic (Figure 1). As expected, the incidence increases with age (Figure 2) and reaches its highest value in the age group between 31 and 35 years (1.21 per 1000 at the national level), with a similar trend in all regions. In terms of the prevalence, it was estimated that 1,889,983 cases were prevalent during the period 2011–2020, resulting in a prevalence rate of 14.0 per 1000 over the same period. The age-specific prevalence rates follow an increasing trend, reaching a peak at 48 years of age.

**Figure 3:**
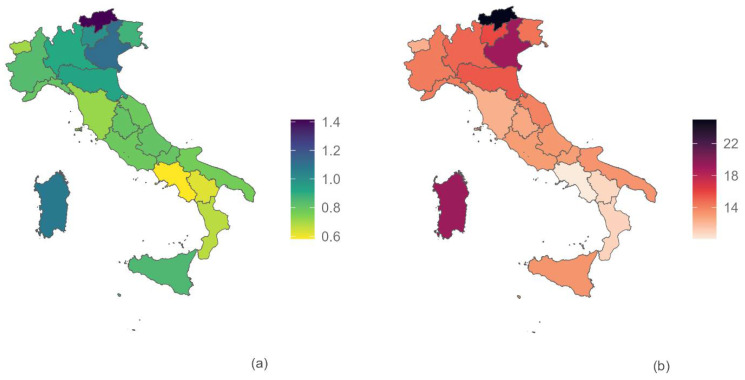
Regional geographical distribution of incidence (**a**) and prevalence (**b**) rates, years 2011–2020.

Incidence rates are not homogeneous across the Italian regions (Table 2). Over the years, a north–south gradient is observed, with generally higher rates in the northern regions (0.944 per 1000, 0.881 in the northwest and 1.030 in the northeast) compared to those in central (0.765 per 1000) and southern Italy (0.373 per 1000) The islands, however, deviate from this pattern, displaying an incidence rate (0.906 × 1000) similar to that of the northern regions. All regions exhibit a decreasing trend, with a more significant decline in Italy’s northern and central regions. As expected, the same north–south geographical gradient is identifiable in the prevalence rates (Figure 3).

### Sensitivity Analysis

The comparison between the incident cases identified by the NHDB and those identified in FVG and Puglia, which integrated the Regional Hospital Discharge Database with the pathological anatomy register, shows that, when the information on pathological anatomy is added, higher estimates of 10% in FVG and 17% in Puglia are obtained (2225 vs. 2441 incident cases identified in FVG; 7189 vs. 8388 incident cases identified in Puglia).

## 4. Discussion

### 4.1. Principal Results

In this national retrospective, population-based study, it was possible to quantify the number of hospitalizations diagnosed with EMS in Italy for the first time. In 2011–2020, 112,945 Italian women had a first diagnosis of this condition, for an overall incidence rate of 0.84 per 1000. Over the years, the values have decreased in almost all Italian regions. A north–south regional gradient was observed. The peak of incident cases was found in the 31–35 age group, and it was estimated that 14 out of 1000 women receive a diagnosis of EMS during childbearing age.

### 4.2. Strengths and Limitations

This is the first study that attempts to estimate the incidence and prevalence of EMS cases in Italy based on a single data source of hospitalizations, which has been available for a long period throughout the country. The main strength of this study is the use of an administrative database available at national level, which allowed us to estimate the incidence and prevalence of EMS in Italy without differences in methods and definitions, to evaluate the time trend and to compare the data by region.

The estimates provided are certainly an underestimate of the real burden of this pathology. In fact, it should be considered that administrative databases, such as the hospital discharge records database, only allow for identifying a portion of the EMS cases. Furthermore, the data source does not allow for evaluating the other characteristics of women, such as socio-economic conditions or obstetric and medical history.

### 4.3. Interpretation and Comparison with Prior Work

The accurate evaluation of the real incidence and prevalence of EMS remains a difficult task. The use of hospital data at national level allows to produce estimates without differences in methods and definitions, to evaluate time trends, and to compare regional data, but only identifies a limited number of cases. Several cases may not require hospitalization or receive a formal diagnosis, especially in young women, in cases of good responses to hormonal treatment [14,15]. Furthermore, non-invasive diagnostic techniques, such as ultrasound and magnetic resonance imaging, may allow outpatients to diagnose the condition [16]. The reliability of these imaging techniques is becoming increasingly high, and their use has been incorporated into more recent guidelines [4,12]. Only the active search for the disease in unselected samples of women of reproductive age (i.e., using questionnaires investigating the presence of typical symptoms, followed by an adequate diagnostic path) can lead to accurate estimates of the prevalence and incidence of EMS. The study by Ferrero et al. suggests that about six out of ten cases of EMS had not been identified before an active search for the disease [17]. A small part of this gap can be filled through data linkage systems, as we saw in FVG and Puglia regions when data from the pathological anatomy records were added. Unfortunately, this data linkage is not possible for Italy on a national level.

Our estimates are based on the decision to assess them in women of reproductive age (between 15 and 50 years). This decision was made for two reasons: (1) EMS affects women mainly at a fertile age. Symptoms are in fact strongly linked to the menstrual cycle, a period in which the endometriotic tissue located outside the endometrium bleeds, causing swelling and inflammations; and (2) to avoid the problem of possible overlap with the diagnosis of adenomyosis. Our national estimate is consistent with the regional [10,18] and international estimates from other countries using the same data sources and age groups [6,19].

An interesting result is the decreasing time trend of EMS diagnosed in hospitals. The decreasing time trend of incidence and prevalence of EMS was also found in other countries [8,20,21]. This phenomenon can be explained by clinical reasons, i.e., the improvement in non-invasive diagnostic methods [4], with a consequent reduction in the number of hospital admissions related to the need for diagnosis, or the reduction in the use of laparoscopy in the diagnosis of infertility and the consequent reduction in the diagnosis of asymptomatic cases [22]. Furthermore, in Italy, increased attention was paid to EMS over the years at the political, cultural and clinical levels: several Italian regions (i.e., Puglia, Campania, Friuli Venezia Giulia) set up observatories of EMS; since 2017, EMS was introduced into the Italian Essential Levels of Assistance (LEA), and included in the list of chronic and disabling diseases; the Italian Ministry of Health funded projects to evaluate incidence and pathogenic mechanisms of the disease; national on-line training initiatives for health professionals have been developed by the Italian National Agency for Regional Healthcare Services (AGENAS), together with awareness-raising initiatives for women [23]; and from 2014, a Word Endometriosis Day was established, with awareness initiatives carried out throughout the country. The increased knowledge and awareness of the disease may have led to its earlier recognition by health professionals and to an early start of hormonal therapy without hospital access.

In Italy, the geographical distribution of EMS shows a clear north–south gradient. This phenomenon has already been seen for other health conditions (i.e., the coverage of mammography or colorectal cancer screening) [24], and can be due to underdiagnoses of the disease in the south region. Part of the explanation could be in the possible differentiated attention by regional governments and health services (which are regional in Italy), and in the imbalance of the available resources (i.e., health spending per capita shows a similar north–south gradient, with lower resources available in southern Italian regions [25]). The greater presence of the private healthcare sector and the lower presence of specialised EMS centres in southern Italian regions may further explain the loss of cases. To this, the effect of a different geographic structure of fertility in Italy could be added, which sees women residing in the central-northern regions having, on average, a higher age at childbirth and a greater number of children, especially in the early years of analysis [17]. Another hypothesis that deserves further investigation is that this gradient could also partly be explained by the role of environmental factors. The literature reports growing evidence of association between environmental pollutants and EMS [26,27,28], including a study in Italy [18]. Indeed, we should note that when discussing our results on the north–south gradient of endometriosis incidence, the northern regions in Italy are known to have higher levels of environmental pollution. This qualitative association must be taken cautiously, and further studies should address this issue at a fine geographical scale and individual level.

## 5. Conclusions

This study allowed for estimating the presence of approximately 176,000 women with a confirmed diagnosis of EMS in Italy. The number rises further when considering the limitations of this study, which only counts cases intercepted by hospital admissions. To assess the extent of this underestimation, an active search for the disease in unselected samples of women of reproductive age is needed. This activity is ongoing in the areas of three Italian regions (FVG, Toscana and Puglia) [17]. However, according to the hypothesis that only one-third of women with EMS reach a confirmed diagnosis [17], in Italy, there would be more than 500,000 women with this condition, demonstrating its significant burden on the population. On the other hand, the study of hospital admissions can provide information about the provision of healthcare services, and analysis using administrative data can be useful for improving healthcare planning in different regions.

Future developments in the analysis of the hospital discharge records database may include assessing the spatial distribution of EMS within each region, so as to determine whether there are high-risk areas associated with specific environmental exposure profiles.

## Figures and Tables

**Table 1:** Codes for the selection of endometriosis in hospital discharge records.

ICD-9-CM Diagnosis Code		ICD-9-CM Procedure Code
617 or 617.0 or 617.1 or 617.2 or 617.3 or 617.4 or 617.5 or 617.6 or 617.8 or 617.9	AND	34.81 or 37.34 or 45.23 or 45.24 or 45.26 or 45.62 or 45.72 or 45.76 or 45.94 or 46.04 or 46.11 or 46.21 or 47.01 or 47.19 or 47.99 or 48.62 or 48.63 or 48.69 or 48.82 or 49.39 or 51.23 or 54.11 or 54.12 or 54.19 or 54.21 or 54.23 or 54.24 or 54.4 or 54.51 or 54.59 or 54.73 or 55.51 or 56.74 or 56.99 or 57.32 or 57.6 or 57.99 or 59.00 or 59.02 or 59.03 or 65.01 or 65.12 or 65.13 or 65.23 or 65.24 or 65.25 or 65.29 or 65.31 or 65.41 or 65.49 or 65.51 or 65.52 or 65.53 or 65.54 or 65.61 or 65.62 or 65.63 or 65.74 or 65.79 or 65.81 or 65.89 or 65.91 or 65.99 or 66.19 or 66.29 or 66.39 or 66.4 or 66.51 or 66.52 or 66.61 or 66.62 or 66.63 or 66.69 or 67.39 or 67.4 or 68.12 or 68.13 or 68.14 or 68.15 or 68.16 or 68.29 or 68.39 or 68.41 or 68.49 or 68.51 or 68.59 or 68.61 or 68.69 or 68.71 or 68.9 or 69.19 or 69.29 or 70.32 or 70.77 or 86.3 or 87.83 or 97.83

**Table 2:** Endometriosis incidence rate per 1000 women aged 15–50 years by region from the 2011–2020 period.

Region	Incidence Annual Rate ×1000										Total 2011–2020		
	2011	2012	2013	2014	2015	2016	2017	2018	2019	2020	*n*	Rate	95% CI
Piemonte	1.064	1.022	0.878	0.937	0.841	0.771	0.688	0.766	0.835	0.540	7810	0.840	(0.822–0.859)
Valle D‘Aosta	0.549	0.587	1.247	0.876	0.679	0.657	0.746	0.495	0.775	0.395	194	0.705	(0.609–0.812)
Lombardia	1.118	1.068	1.001	0.942	0.941	0.930	0.945	0.856	0.687	0.597	20,126	0.912	(0.900–0.925)
Liguria	0.964	1.051	0.958	0.853	0.721	0.778	0.758	0.532	0.754	0.561	2504	0.801	(0.770–0.833)
**Northwest**	**1.085**	**1.050**	**0.966**	**0.932**	**0.892**	**0.872**	**0.859**	**0.801**	**0.732**	**0.577**	**30,634**	**0.881**	**(0.871–0.891)**
PA Bolzano	1.677	1.867	1.762	1.688	1.651	1.374	1.158	1.014	0.997	0.884	1706	1.414	(1.348–1.483)
PA Trento	1.259	1.051	1.149	1.212	1.001	0.888	0.797	0.943	0.993	0.689	1198	1.002	(0.946–1.060)
Veneto	1.208	1.212	1.144	1.159	1.211	1.182	1.035	1.060	1.049	0.879	12,085	1.117	(1.098–1.138)
Friuli-Venezia Giulia	1.074	0.952	0.882	0.864	0.830	0.921	0.799	0.808	0.905	0.695	2225	0.876	(0.840–0.913)
Emilia Romagna	1.017	1.059	0.972	1.015	0.972	0.930	0.934	0.840	0.839	0.672	8963	0.928	(0.909–0.947)
**Northeast**	**1.147**	**1.151**	**1.082**	**1.102**	**1.093**	**1.055**	**0.968**	**0.943**	**0.949**	**0.772**	**26,177**	**1.030**	**(1.017–1.042)**
Toscana	0.938	0.885	0.804	0.791	0.683	0.607	0.596	0.668	0.646	0.495	5732	0.716	(0.698–0.735)
Umbria	0.837	0.981	1.001	0.976	0.901	0.722	0.736	0.661	0.651	0.492	1532	0.802	(0.763–0.844)
Marche	0.887	0.815	0.931	0.865	0.779	0.649	0.808	0.763	0.748	0.572	2616	0.785	(0.755–0.816)
Lazio	0.980	0.937	0.869	0.803	0.790	0.721	0.721	0.699	0.661	0.629	10,346	0.784	(0.769–0.799)
**Centre**	**0.945**	**0.909**	**0.867**	**0.820**	**0.764**	**0.678**	**0.695**	**0.695**	**0.667**	**0.572**	**20,226**	**0.765**	**(0.754–0.776)**
Abruzzo	0.780	0.872	0.916	0.830	0.888	0.802	0.743	0.860	0.746	0.584	2355	0.805	(0.773–0.838)
Molise	0.742	0.910	0.774	0.686	0.742	0.907	0.776	0.655	0.673	1.145	538	0.799	(0.733–0.869)
Campania	0.633	0.659	0.650	0.609	0.586	0.551	0.587	0.532	0.557	0.429	8155	0.582	(0.569–0.594)
Puglia	0.801	0.800	0.802	0.785	0.823	0.821	0.743	0.776	0.724	0.578	7189	0.768	(0.750–0.786)
Basilicata	0.571	0.849	0.706	0.649	0.616	0.574	0.683	0.608	0.513	0.442	799	0.625	(0.582–0.670)
Calabria	0.692	0.755	0.756	0.664	0.728	0.653	0.704	0.650	0.605	0.487	3031	0.673	(0.649–0.697)
**South**	**0.702**	**0.744**	**0.737**	**0.690**	**0.705**	**0.673**	**0.669**	**0.652**	**0.628**	**0.508**	**22,067**	**0.673**	**(0.665–0.682)**
Sicilia	0.955	0.923	0.847	0.863	0.909	0.833	0.849	0.781	0.867	0.659	9899	0.851	(0.835–0.868)
Sardegna	1.174	1.219	1.147	1.056	1.176	1.122	1.085	1.012	0.945	0.812	3942	1.081	(1.048–1.115)
**Islands**	**1.008**	**0.994**	**0.919**	**0.909**	**0.972**	**0.902**	**0.905**	**0.835**	**0.885**	**0.695**	**13,841**	**0.906**	**(0.891–0.921)**
**ITALY**	**0.966**	**0.960**	**0.907**	**0.880**	**0.868**	**0.823**	**0.806**	**0.775**	**0.753**	**0.610**	**112,945**	**0.839**	**(0.834–0.844)**

## Data Availability

The datasets generated and analyzed during the current study are not publicly available due to privacy regulations.

## Acronyms

EMS = endometriosis, FVG = Friuli-Venezia Giulia Region, NHDB = National Hospital Discharge Database, ICD-9-CM = International Classification of Diseases, Ninth Revision Clinical Modification, CI = confidence interval.
